# Literary Notes

**Published:** 1908-07

**Authors:** 


### LITERARY NOTES.

The American Jour. of Obstetrics and Diseases of Women and Children has stood the test of time and for the past forty years it has been recognised at home and abroad as the leading authority on the subjects of Gynecology, Abdominal Surgery, Obstetrics and Pediatrics. The publisher, Messrs William Wood & Company, 51 Fifth Avenue, New York, offer to send free of charge on request the June, July and August, 1908, issues, or the July, August and September, 1908, issues. Be sure to specify which three are preferred.

---

The State Education Building is the title of a brochure recently issued by the New York State Department of Education, Albany, N. Y. It contains a complete description of the plans of the new building soon to be constructed, the foundation of which will soon be laid. It will be one of the finest structures devoted strictly to education administration in the world. The drawings are indicative of a remarkable degree of perfection, of which every citizen should be proud.

---

School Hygiene is the name of a new journal which, according to its editorial announcement, represents an effort to place before the public facts and propositions in the movement to secure improved school conditions for children; also to awaken public interest in the importance of the subject. It will vary from eight to sixteen pages, double column quarto, in the amount of reading matter. It is published under the auspices of the American School Hygiene Association, Boston, and is edited by Dr. George S. C. Badger, 48 Hereford Street, Boston, Mass. Contributions to its columns are earnestly solicited.

